# Heritable human genome editing and the politics of law: the South African case study

**DOI:** 10.1093/jlb/lsaf029

**Published:** 2025-12-23

**Authors:** Donrich Thaldar, Sheetal Soni, Larisse Prinsen, Ntokozo Mnyandu, Marietjie Botes, Julian Kinderlerer

**Affiliations:** School of Law, University of KwaZulu-Natal, Mazisi Kunene Road, Glenwood, Durban, South Africa; School of Law, University of KwaZulu-Natal, Golf Road, Scottsville, Pietermaritzburg, South Africa; Faculty of Law, University of the Free State, Nelson Mandela Drive, Park West, Bloemfontein, South Africa; School of Law, University of the Witwatersrand, Jan Smuts Avenue, Braamfontein, Johannesburg, South Africa; Centre for Research on Evaluation, Science and Technology, Stellenbosch University, Ryneveld Street, Stellenbosch, South Africa; Faculty of Law, University of Cape Town, Cross Campus Road, Cape Town, South Africa

**Keywords:** constitutional rights, ethical oversight, heritable human genome editing, National Health Research Ethics Council, public health, South Africa

## Abstract

Heritable human genome editing (HHGE) has emerged as one of the most contested frontiers of bioscience, where law is mobilized as a political instrument. This article uses South Africa as a case study in the politics of law in HHGE governance, showing how even a substantively strong regulatory framework can collapse when procedurally fragile and politically contested. The National Health Research Ethics Council’s 2024 guidelines established a regulated pathway for HHGE research and anticipated the eventual possibility of clinical application. The guidelines were aligned with constitutional rights and contained various ethical safeguards. Yet their adoption was procedurally weak: consultation was confined to research ethics committees, excluding broader scientific and public engagement. This lack of participatory legitimacy left the guidelines vulnerable. Critics seized on the fact that the guidelines contemplated children being born with edited genomes, collapsing anticipation into permission. These critiques were amplified by institutional actors, culminating in the repeal of the guidelines in 2025 and their replacement with an indefinite placeholder. The South African case highlights a lesson of global significance: that the governance of HHGE, and of emerging biotechnologies more broadly, depends not only on substantive ethical safeguards but also on inclusive procedures that can withstand political contestation.

## I. INTRODUCTION

Heritable human genome editing (HHGE) potentially represents one of the most transformative developments in modern biotechnology. It offers the prospect of preventing serious genetic diseases and reshaping how societies address inherited health risks by intervening at the earliest stages of human life.^1^ Yet alongside its promise, HHGE raises profound questions of law, ethics, and governance: How should societies weigh life-saving potential against scientific uncertainty? What responsibilities do regulators bear when technological capabilities outpace political consensus? And who should determine when, and under what conditions, the technology may move from laboratory to clinic?^2^

Globally, debate over HHGE has been framed above all in ethical terms. Three recurring themes dominate: beneficence and non-maleficence, asking whether the ability to cure or prevent genetic disease justifies unknown risks;^3^ justice and equity, questioning whether HHGE will exacerbate inequality or, alternatively, broaden access to health;^4^ and autonomy, concerning the scope of reproductive freedom and principled limits on parental choice.^5^ These themes provide an essential backdrop, but the dynamics of governance have often turned less on sustained ethical engagement than on how legal texts and constitutional guarantees are interpreted—or misinterpreted—in ways that advance particular political positions. In this sense, HHGE has become not only a scientific and ethical frontier but also a field in which law itself functions as a tool of persuasion and authority.

While HHGE holds transformative promise, its scientific maturity remains limited. Current methods are far from achieving safe and efficient germline editing in humans.^6^ Research in animal models has begun to explore possibilities such as heritable resistance to infectious diseases—including human immunodeficiency virus (HIV) and tuberculosis (TB)—but these remain speculative and technically remote.^7^ Advances in base editing, prime editing, and experimental in utero delivery highlight potential directions, yet major hurdles of efficiency, safety, and precise targeting persist.^8^ For now, clinical application is not imminent.

It is against this ethical and scientific backdrop that South Africa briefly emerged as a significant case study. In May 2024, the National Health Research Ethics Council (NHREC) adopted guidelines establishing a regulatory pathway for HHGE research, including the possibility of clinical trials, subject to stringent safeguards.^9^ Substantively, the guidelines were robust, embedding safeguards such as compelling scientific justification, ministerial authorization, and long-term monitoring.^10^ At the same time, they left certain questions underdeveloped—for instance, how to define sufficient safety, identify relevant stakeholders, or structure ministerial discretion—issues that called for further elaboration even though they did not drive the controversy that ultimately engulfed the framework. Procedurally, however, the guidelines were fragile: consultation had been confined largely to research ethics committees, excluding geneticists, other research groups working on the ethical and legal dimensions of HHGE, and the broader public. This lack of inclusivity left the guidelines politically vulnerable. When academic criticism later focused on the fact that the guidelines contemplated the eventual possibility of children being born with edited genomes, those critiques gained traction and ultimately contributed to the NHREC’s decision to repeal the guidelines in July 2025.^11^

This article reflects on that episode, offering both a legal analysis and a diagnosis of the political dynamics that surrounded it. We argue that the NHREC’s 2024 guidelines represented a thoughtful and ethically robust framework, aligned with South Africa’s constitutional values^12^ and evidence of public support^13^ but that their procedural weakness rendered them institutionally fragile. By contrast, the critiques advanced against the guidelines relied less on substantive legal or ethical reasoning than on rhetorical strategies that misrepresented the law, disregarded embedded safeguards, and marginalized South African public opinion. More broadly, the South African case illustrates how global debates over HHGE are shaped not necessarily by rational analysis but by politics—whose voices are amplified, whose interpretations are treated as authoritative, and how law is mobilized to secure those positions.

We proceed as follows. First, we set out the South African context, including the health-care realities and the statutory and constitutional framework within which HHGE must be regulated. Second, we analyze the NHREC’s short-lived 2024 guidelines,^14^ demonstrating their ethical safeguards and legal coherence. Third, we critically assess the major academic interventions opposing the guidelines, situating them within broader political currents. Finally, we turn to the events leading to the repeal of the guidelines, offering a critique of both the process and the wider lessons for HHGE governance worldwide.

## II. BACKGROUND

Any evaluation of South Africa’s short-lived experiment with regulating HHGE must be grounded in the country’s distinctive legal, social, and health realities. South Africa is not simply a passive recipient of global bioscience debates but a jurisdiction with its own constitutional framework, statutory regime, and pressing public health priorities.^15^ This section therefore sets the stage for analysing the NHREC’s 2024 guidelines by first examining the relevant provisions of South African law,^16^ then situating HHGE against the backdrop of the country’s ongoing health crises—most notably the twin epidemics of TB and HIV/acquired immunodeficiency syndrome (AIDS)—and finally considering what is known about South African public opinion on genome editing.^17^

### II.A. South African Law on HHGE

#### II.A.1. The ban of human reproductive cloning

The lawfulness of HHGE in South Africa has been a point of contention,^18^ with the debate centering on section 57(1) of the National Health Act (NHA).^19^ The section provides:

57.(1) A person may not—

- manipulate any genetic material, including genetic material of human gametes, zygotes, or embryos; or
- engage in any activity, including nuclear transfer or embryo splitting,

for the purpose of the reproductive cloning of a human being.^20^

The first interpretive question is whether the prohibition on manipulating genetic material in subsection (a) applies only to the explicitly listed examples—human gametes, zygotes, and embryos. A narrow reading of this kind is unlikely. The Constitutional Court has clarified that the term ‘including’ is illustrative rather than exhaustive.^21^ The listed items are therefore examples of ‘any genetic material’, not limitations on its scope.

A second question concerns the meaning of ‘manipulate’, which is not defined in the NHA. It must therefore be given its ordinary meaning, such as ‘to manage or utilize skillfully’^22^ or ‘to use something with a lot of skill’.^23^ Under this broad understanding, manipulating genetic material encompasses activities such as handling, controlling, or utilizing DNA. Routine research practices in South Africa—such as DNA isolation or genetic sequencing—fall within this definition, as does *in vitro* fertilization (IVF), which involves skillful handling of gametes and embryos. Taken literally, these commonplace practices would appear to constitute manipulation of genetic material. Does this mean that IVF, DNA isolation, or genetic sequencing is unlawful in South Africa?

The answer is no, because of the qualifying phrase at the end of section 57(1): ‘for the purpose of the reproductive cloning of a human being’. ^24^ This phrase makes it clear that manipulation of genetic material is unlawful only when performed for the specific purpose of reproductive cloning. Manipulation for other purposes—whether routine IVF, DNA isolation, or genetic sequencing—falls outside the scope of the prohibition.

It is important to note the structural placement of this qualifying phrase. First, it is separated with a line break from subsections (a) and (b). Second, it is aligned with the main body of section 57(1), not indented like subsections (a) and (b). In statutory interpretation, such visual cues matter: courts often regard line breaks, indentation, and formatting as part of the text’s syntax, shaping whether a phrase attaches only to the immediately preceding subsection or to the provision as a whole.^25^ The structural placement here indicates that the qualifying phrase is not limited to subsection (b) but applies equally to subsection (a). Accordingly, the prohibition on manipulating genetic material in subsection (a) is restricted to instances where the manipulation is undertaken for reproductive cloning.

It follows that section 57(1) does not outlaw HHGE. HHGE is not equivalent to the reproductive cloning of a human being, which is the specific conduct prohibited by the provision. Reproductive cloning involves producing a genetic duplicate of an existing individual by nuclear transfer, with the result that the clone shares an identical nuclear genome with the donor.^26^ By contrast, HHGE does not replicate identity; it alters the genome of an existing embryo, producing a child who is genetically unique. The purposes are likewise distinct: cloning aims at genetic replication, whereas HHGE aims at prevention of genetic disease, immunity against communicable disease, and potentially, enhancement. This conceptual distinction reinforces the conclusion that section 57(1) does not impose a blanket prohibition on genetic manipulation of genetic material, thus leaving open a legal pathway for HHGE research.

#### II.A.2. Section 57(4) and the 14-day rule

Section 57(4) of the NHA provides:

The Minister may permit research on stem cells and zygotes which are not more than 14 days old on a written application and if—

- the applicant undertakes to document the research for record purposes; and
- prior consent is obtained from the donor of such stem cells or zygotes.^27^

The philosophical basis of the 14-day rule is well established. Originating with the Warnock Committee in the UK and subsequently adopted in many jurisdictions, it reflects a compromise between recognizing the scientific value of early embryo research and acknowledging the moral value that many in society attach to embryos.^28^ The concern is that research on embryos *in vitro* often entails their destruction. The 14-day line marks a threshold beyond which the embryo begins to develop the primitive streak and is seen as acquiring heightened moral status.^29^ South African law already recognizes the principle of graded moral protection of unborn life. For example, the Choice on Termination of Pregnancy Act permits termination in the first trimester on demand but increasingly restricts it in the second and third trimesters, reflecting a growing state interest in protecting foetal life.^30^ The Constitutional Court has acknowledged the legitimacy of this legislative approach, recognizing both the pregnant woman’s rights and the state’s duty to protect potential life.

In this light, section 57(4) functions as a carefully calibrated carve-out. It permits preclinical research on early embryos—zygotes up to 14 days—under ministerial authorization, balancing the freedom of scientific research with protection of developing life. The mischief it targets is clear: preventing destructive experimentation on embryos that have developed beyond the stage regarded as morally acceptable.

The more difficult question is whether this limitation extends to clinical trials in which an edited embryo is transferred in utero and allowed to develop beyond 14 days, potentially resulting in the live birth of a genetically edited child. On a broad reading, such trials might be construed as embryo research continuing beyond the 14-day period, thus falling foul of section 57(4). However, such a reading is misplaced.

HHGE clinical trials conceptually move away from embryo research toward human-participant research. Once an edited embryo is transferred in utero, it no longer functions as an isolated legal object of study. While biologically distinct, the law treats the embryo as subsumed within the pregnant woman’s legal subjecthood. From that point onward, research is not ‘embryo research’ in the sense contemplated by section 57(4)^31^ but research on human participants. A simple illustration shows why this conceptual shift matters. Suppose researchers conducted a non-invasive imaging study—say, high-resolution, real-time 3-dimensional ultrasound imaging (4D ultrasound)—on an implanted embryo at a stage well beyond 14 days. Clearly, the proper regulatory framework for such a study is not the 14-day rule but the human-participant regime: the pregnant woman’s informed consent, ethics approval, and the protections associated with research on living persons. It would be artificial, if not absurd, to characterize such an observational study as prohibited embryo research under section 57(4). The same logic applies to HHGE clinical trials: once implantation occurs, the embryo is no longer a laboratory object of destructive research but part of the pregnant woman’s legal subjecthood.

Section 71 of the NHA provides the foundation for this framework.^32^ At the intervention stage, subsection 71(1) applies: research or experimentation on a living person may only be conducted in the prescribed manner and with the pregnant woman’s informed consent.^33^ Once a child is born, the ongoing monitoring and follow-up that are integral to HHGE trials shift into research on a minor. Here subsections 71(2) and (3) become relevant: if the intervention is therapeutic in purpose, research may proceed only if it is in the best interests of the child, subject to prescribed conditions, and with the consent of the parents or guardians.^34^ If the intervention is non-therapeutic, stricter safeguards apply: in addition to parental consent, ministerial consent is required.^35^ In both scenarios, the child’s consent is not applicable at the outset, as the child does not yet exist as a legal subject when the research begins, but once the child develops sufficient capacity, assent obligations may arise for ongoing participation.

Section 72 complements this regime by empowering the NHREC to set norms and standards for clinical trials, thereby ensuring consistent ethical oversight.^36^ The Regulations Relating to Research with Human Participants reinforce this framework by requiring in regulation 2(a) that health research involving human participants must, at a minimum, comply with the NHREC’s national ethical guidelines.^37^ Accordingly, implantation of an edited embryo without first obtaining ethics approval for a clinical trial under the NHREC guidelines would be unlawful. Taken together, these provisions show that HHGE clinical trials are not captured by section 57(4)’s prohibition but are instead subject to a robust legal framework for human subject research—one that protects the pregnant woman’s rights and the welfare of the resulting child.

The decisive point is the mischief at which section 57(4) is aimed. Its purpose is to prevent destructive *in vitro* research on embryos beyond 14 days—research that extinguishes rather than sustains developmental potential once a morally significant threshold is crossed. HHGE clinical trials are the inverse: their very purpose is to support implantation and continued development *in utero*. Extending the 14-day prohibition to such trials would therefore misapply the provision, collapsing its rationale and rendering incoherent the careful balance struck by the Act. The appropriate safeguards are instead those that protect human participants: section 71’s requirements for consent and best-interest determinations,^38^ section 72’s mandate for NHREC oversight,^39^ and the Regulations binding researchers to national ethical guidelines.^40^ Properly understood, then, section 57(4) is not a barrier to HHGE clinical trials.

### II.B. The South African Health Landscape

Any discussion of HHGE in South Africa must be situated within a health context that looks strikingly different from that of the global north. Whereas Europe and North America face mortality dominated by non-communicable diseases (NCDs) such as cancer and cardiovascular disease, ^41^ South Africa continues to confront the dual epidemics of TB and HIV/AIDS. TB remains the leading cause of natural death in South Africa, responsible for roughly 56,000 deaths in 2023, with nearly half of these cases co-infected with HIV.^42^ HIV prevalence is among the highest globally: about 8.5 million people—13.9% of the population, and nearly one in five adults aged 15–49—live with HIV.^43^ Although expanded antiretroviral treatment programs have reduced annual AIDS-related deaths to around 49,000, HIV/AIDS remains one of the country’s principal causes of mortality.^44^ These twin epidemics keep life expectancy at an average of about 63 years (60 for men, 65 for women), far below that of the USA (77 years) or the UK (80 years).^45^

Comparative data underscore the severity of this landscape. South Africa’s TB incidence is about 427 new cases per 100,000 annually, among the highest in the world; by contrast, the rate is 195 in India, 219 in Nigeria, 49 in Brazil, 8 in the UK, and 3 in the USA.^46^ Similarly, adult HIV prevalence is nearly 17% in South Africa, compared to 0.2% in India, 0.6% in Brazil, 1%–2% in Nigeria, 0.4% in the USA, and 0.2% in the UK.^47^ South Africa’s maternal mortality ratio, at 127 deaths per 100,000 live births, is significantly higher than in the global north (21 in the USA and 10 in the UK) but markedly lower than in Nigeria (over 1000).^48^ Infant mortality rates (≈24 per 1000 live births) similarly exceed those of wealthier nations (typically <5 per 1000).^49^ These disparities make clear that the challenges South Africa faces are more comparable to those of other high-burden, middle-income contexts than to those of Western nations.

Alongside the infectious disease crisis, South Africa faces a rising burden of NCDs such as diabetes (now the second leading cause of natural death), hypertension, stroke, and cancer.^50^ The result is a ‘double burden’ of disease: hospitals often treat patients with advanced TB or HIV in one ward and patients with chronic metabolic disease in another. This co-existence of ‘developing world’ and ‘developed world’ disease profiles makes the South African context distinctive. It also highlights the appeal of innovative health interventions: a country that has led the world in scaling up antiretroviral therapy (with nearly 6 million people now on treatment) and in rolling out novel TB diagnostics and therapies has also demonstrated openness to exploring cutting-edge technologies.^51^ South African researchers are already pursuing somatic gene-editing approaches to HIV, TB, and hepatitis B, including experimental clustered regularly interspaced short palindromic repeats (CRISPR)-based disruption of viral pathways.^52^ Evidence from immunogenetics further reinforces this rationale: for TB, host genetic variation in human leukocyte antigen (HLA) and cytokine pathways influences susceptibility and immune control,^53^ while in HIV, CCR5 mutations and HLA polymorphisms shape vulnerability and disease progression.^54^ At the same time, gene-editing approaches for infectious diseases remain at an early stage worldwide, with significant translational hurdles before they could offer public health impact.^55^

Finally, genetic and congenital disorders present another layer of health challenge. Modeling suggests that roughly 32,000 South African children are born each year with serious congenital conditions, and rare diseases collectively affect about 4 million South Africans, with approximately 72% of which are genetic.^56^ Conditions such as oculocutaneous albinism are relatively common, while other disorders, such as porphyria or familial hypercholesterolemia, are concentrated in specific population groups.^57^ These burdens, coupled with gaps in screening and treatment capacity, create an additional rationale for considering the future role of genome editing. In sum, South Africa’s health landscape is defined less by the non-communicable disease burdens that dominate in the global north and more by infectious disease epidemics and maternal–child health challenges that it shares with other low- and middle-income countries (LMICs) such as India, Brazil, and Nigeria. This epidemiological profile highlights why questions of biomedical innovation, including HHGE, resonate differently in South Africa: the prospect of interventions that could reduce vulnerability to TB or HIV is situated against pressing public health crises, not only theoretical concerns.

### II.C. Constitutional Rights Relevant to HHGE

#### II.C.1. Human dignity and the right of access to healthcare

Two constitutional provisions are central to South Africa’s approach to biomedical innovation: section 10, which guarantees the right to dignity, and section 27, which guarantees the right to have access to healthcare services.^58^ The Constitutional Court has emphasized that these rights are interdependent: dignity underpins the recognition that all individuals have equal moral worth, while section 27 imposes concrete duties on the state to progressively realize access to healthcare in ways that affirm that worth.

The Treatment Action Campaign (TAC) case provides the most important judicial exposition of these rights in the healthcare context.^59^ The litigation was triggered by government policy restricting the use of the antiretroviral drug Nevirapine, which was shown to significantly reduce mother-to-child transmission of HIV. Instead of permitting public hospitals and clinics broadly to use the drug, the government confined access to a limited number of ‘pilot sites’.^60^ This decision excluded the overwhelming majority of pregnant women and their babies from a safe, effective, and affordable intervention.

The TAC, a civil society organization, challenged the government’s stance as unconstitutional, arguing that it violated the rights to life, dignity, and access to healthcare. The Constitutional Court agreed, finding that the government’s policy was unreasonable under section 27.^61^

The Court’s reasoning established several enduring principles of direct relevance to HHGE:

1. *Progressive realization with reasonableness*. Section 27(2) of the Constitution obliges the state to take ‘reasonable legislative and other measures, within its available resources’, to achieve the progressive realization of access to healthcare.^62^ The Court stressed that ‘reasonableness’ demands flexibility, adaptability, and responsiveness to evidence.^63^ A policy that rigidly excludes a large majority from access to a simple, life-saving measure cannot be reasonable.
2. *Prioritizing the vulnerable*. The Court held that a health programme cannot be deemed reasonable if it excludes a ‘significant segment of society’, particularly those who are indigent and depend entirely on the public health system. This interpretation aligns section 27 with section 9 (equality) and section 10 (dignity):^64^ policies that systematically disadvantage the poor are incompatible with constitutional values.
3. *Negative obligations*. Importantly, the Court reaffirmed that socio-economic rights are not only positive in nature (requiring the state to act) but also impose negative obligations: the state may not act in ways that impede existing or reasonably available access to healthcare. Just as in *Grootboom* the Court held that the state cannot evict people from housing without lawful justification,^65^ so too in *TAC* it held that the state cannot prevent doctors in capable public facilities from providing Nevirapine where it is safe and indicated. This negative duty applies across both the public and private healthcare sectors: government may not erect barriers to access, irrespective of the institutional setting.
4. *Dignity and life*. While the judgment was formally grounded in section 27 and the rights of children,^66^ the Court explicitly acknowledged that denying access to life-saving medicines infringes dignity. It explained that to condemn individuals to ‘a life below a basic level of dignified human existence’ is inconsistent with the foundational values of the Constitution. Earlier in *Soobramoney v. Minister of Health (KwaZulu-Natal)*,^67^ the Court had underscored that socio-economic rights are essential to the restoration of dignity in a society marked by poverty and inequality. *TAC* thus reinforced that access to healthcare is not only a socio-economic entitlement but also a direct manifestation of dignity.

Taken together, the *TAC* principles make clear that health policy in South Africa must facilitate reasonable access, prioritize the vulnerable, and avoid unjustified barriers.

A more recent case, *Surrogacy Advisory Group v. Minister of Health*,^68^ extended these principles into the domain of reproductive health care. The applicants challenged provisions of the Regulations Relating to Artificial Fertilisation of Persons that imposed a blanket prohibition on pre-implantation sex selection for non-medical reasons. The High Court held that such restrictions infringed section 27’s guarantee of access to reproductive health care, which is expressly encompassed within the right to healthcare services, and also violated the right to dignity.

Two principles of direct relevance to HHGE emerge from this judgment. First, the Court reaffirmed the negative obligation on the state not to impede access to healthcare technologies, even where the intervention is available only for those who can pay. Once a technology is safe and effective, the government may not impose blanket prohibitions that prevent individuals from exercising reproductive choice. Second, the Court insisted on the moral neutrality of the state: the government cannot justify prohibiting access to health technologies based on contested moral views. In striking down the prohibition on non-medical pre-implantation sex selection, the Court emphasized that the state is not permitted to dictate the moral acceptability of private reproductive decisions.

The implications for HHGE are significant. Should genome editing techniques mature into safe and effective applications within reproductive health care, they would fall squarely under the section 27 right of access to healthcare services, including reproductive health care.^69^ The state would therefore bear not only a positive duty to progressively realize equitable access, as in *TAC*,^70^ but also an immediate negative duty, as in *Surrogacy Advisory Group*,^71^ not to block or restrict access on moralistic grounds. In short, South Africa’s constitutional framework requires that new biomedical technologies be evaluated on evidence-based grounds rather than on the shifting sands of political or moral controversy.

#### II.C.2. Freedom of scientific research: the ‘Cinderella right’

Beyond dignity and healthcare, the Constitution also protects freedom of scientific research under section 16(1)(d), housed within the broader right to freedom of expression.^72^ Thaldar and Steytler describe this as the Constitution’s ‘Cinderella right’—acknowledged in the text but largely forgotten in constitutional discourse and judicial practice.^73^ No Constitutional Court judgment has yet directly invoked it, but its inclusion in the Constitution is both deliberate and significant.

To understand the scope of this right, it must be read in the context of South African jurisprudence on freedom of expression. The Constitutional Court has consistently explained that expression is protected because it serves several foundational purposes:

1. *Truth-seeking (marketplace of ideas)*: Free expression enables society to test competing claims and discover knowledge (*South African National Defence Union v. Minister of Defence*).^74^ Suppressing scientific research impedes the discovery of scientific truth in precisely the same way as suppressing dissenting speech.
2. *Democratic participation:* Expression sustains open debate and deliberation in a democratic society (*Islamic Unity Convention v. Independent Broadcasting Authority*).^75^ In the case of scientific research—particularly in controversial fields like HHGE—the democratic significance lies less in directly informing policy than in the act of pursuing inquiry itself. At a time when international movements seek to impose moratoria or suppress such research,^76^ conducting HHGE research is a political act in its own right. It signals resistance to ideological pressures and affirms South Africa’s commitment to openness, autonomy, and democratic self-determination in science policy.
3. *Individual self-fulfillment and creativity*: Freedom of expression protects the autonomy and creativity of individuals (*Laugh It Off Promotions CC v. SAB International*).^77^ Scientific inquiry is a paradigmatic form of intellectual creativity and self-fulfillment.

Freedom of scientific research therefore protects scientists’ autonomy not only to pursue inquiry and generate knowledge but also to engage in the inherently political act of undertaking research in contested fields.^78^ Especially in the case of HHGE, where international calls for moratoria and suppression remain strong,^79^ the very decision to conduct research communicates resistance to external ideological pressures and affirms South Africa’s constitutional identity as an open and democratic society. Section 16(1)(d) does not mean that such research is exempt from regulation^80^; as with other forms of expression, restrictions may be legitimate where they are evidence-based, proportionate, and aimed at protecting competing constitutional rights such as dignity, safety, or equality. But outright prohibitions stand in direct tension with the constitutional commitment to freedom of scientific research.

Thaldar and Steytler emphasize that no South African court has yet interpreted section 16(1)(d) directly, but they argue that existing jurisprudence on freedom of expression points strongly toward a robust understanding of scientific freedom.^81^ For example, the Constitutional Court has insisted that even unpopular or controversial ideas merit protection (*South African National Defence Union v Minister of Defence*)^82^ and that restrictions on expression must be narrowly tailored and strictly justified (*Islamic Unity Convention v. Independent Broadcasting Authority*).^83^ These principles are directly applicable to scientific inquiry. Taken together, they suggest that section 16(1)(d)^84^ secures constitutional space for pioneering and controversial forms of research—including HHGE.

#### II.C.3. Relevance to HHGE

For HHGE, these constitutional rights intersect in two complementary ways.

*Freedom of scientific inquiry*. Section 16(1)(d) affirms the autonomy of scientists to pursue HHGE research.^85^ In the present global climate, where strong voices abroad advocate moratoria or even outright bans,^86^ the very act of undertaking HHGE research in South Africa is itself a political act. It signals a commitment to constitutional democracy by refusing to let ideological currents dictate the boundaries of scientific progress. In this way, the exercise of scientific freedom becomes an expression of South Africa’s identity as an open and democratic society and contributes to the global bioscience-political discourse on HHGE.^87^ Regulation of HHGE may legitimately safeguard competing constitutional rights, such as the bodily integrity of research participants,^88^ but categorical prohibitions or ideologically motivated restrictions are inconsistent with section 16(1)(d).^89^ Protecting the freedom of scientific research thus ensures that South Africa remains a jurisdiction where pioneering, controversial, and politically contested inquiry can be pursued responsibly.^90^

*Access and dignity*. Should HHGE interventions in the future prove safe and effective, the Constitution obliges the state to ensure access.^91^ Crucially, HHGE would be used as part of reproductive decision-making: prospective parents might wish to use genome editing to, for example, prevent their children from inheriting a genetic disorder, or even to reduce susceptibility to endemic diseases such as HIV or TB. In this respect, HHGE would be best understood as a form of assisted reproductive technology, placing it squarely within the scope of ‘reproductive health care’ protected by section 27.^92^

The Constitutional Court in *Treatment Action Campaign* established that section 27 imposes a positive duty on the state to progressively realize access to health care, especially in relation to serious conditions, and an immediate negative duty not to impede existing or available access.^93^  *Surrogacy Advisory Group v Minister of Health* extended these principles specifically to reproductive technologies, striking down a ban on non-medical pre-implantation sex selection.^94^ The Court held that the state must remain morally neutral and may not prohibit reproductive technologies on the basis of contested ethical views. By analogy, once HHGE matures into a safe and effective reproductive option, the state cannot prohibit its use simply because of moralistic objections.

Together, these rights mean that once HHGE yields safe and effective applications, prospective parents who wish to use it would be constitutionally entitled to do so as part of their right to reproductive health care. Importantly, this entitlement extends even to non-health-related purposes, such as selecting for traits like intelligence or athleticism. The analogy here is clear: in *Surrogacy Advisory Group v Minister of Health*, the Court struck down a prohibition on non-medical pre-implantation sex selection, holding that the state may not interfere in reproductive choices on the basis of contested moral views.^95^ By the same reasoning, the state may not impose moralistic restrictions on parental use of HHGE for non-therapeutic purposes, provided the interventions are safe.

At the same time, where HHGE is used for health-related purposes—for example, to prevent inherited disorders or to confer resistance to endemic diseases such as TB and HIV^96^—the Constitution imposes more than a duty of non-interference. In such cases, section 27 requires the state to progressively integrate these technologies into the health system in ways that promote equal access, so that they do not remain confined to a privileged minority.^97^

In short, while non-health applications of HHGE invoke only the negative duty of the state not to impede access, health-related applications invoke both the negative duty not to obstruct and the positive duty to facilitate broader access. This dual structure underscores how the South African Constitution protects reproductive freedom in its broadest sense while also affirming equality and dignity in the realm of public health.

In sum, South Africa’s Constitution offers a distinctive framework for HHGE: it secures the freedom to conduct research in contested fields and ensures that, once proven safe and effective, HHGE may be accessed by prospective parents as a protected form of reproductive health care—shielded both from unreasonable barriers and from moralistic state interference.

### II.D. Public Opinion

Although no nationally representative survey of South Africans has yet been conducted on HHGE, there is nevertheless valuable insight into how the public may view this technology. In 2022, South Africa hosted the first deliberative public engagement study on HHGE in Africa^98^—a process akin to what is often referred to in the United States as a ‘citizens’ jury’. Unlike conventional opinion polls that capture surface-level reactions, this methodology brings together a diverse group of participants, equips them with balanced information, and facilitates structured deliberation.^99^ The result is not simply opinion but considered judgment—an especially important distinction when dealing with complex and unfamiliar scientific technologies.^100^

The outcomes of the South African study were striking, and they tie directly to the country’s unique health landscape. Once participants had deliberated, an overwhelming majority supported the use of HHGE to prevent serious inherited conditions—provided the technology is safe and effective.^101^ Even more notable, given South Africa’s public health realities, was the strong support for HHGE directed at conferring immunity against TB and HIV/AIDS. Nearly all participants endorsed such applications, provided the technology could be shown to be safe and effective. This resonates with the fact that TB remains South Africa’s leading cause of death and that HIV continues to affect millions—burdens largely absent from the experience of populations in Europe or North America.^102^ The contrast illustrates how local epidemiological realities shape public attitudes toward emerging biotechnologies. At the same time, participants were firmly opposed to using HHGE for enhancement purposes such as altering intelligence, appearance, or personality traits.^103^ They also emphasized that HHGE should be accessible as a healthcare service rather than an elite privilege.^104^

Placed in a global perspective, these findings are consistent with a broader international pattern: publics across the world tend to be more supportive of HHGE when it is used to prevent disease than when it is proposed for enhancement. For instance, a 2017 Royal Society survey in the UK found that 76% of adults were positive about correcting an inheritable disorder through genome editing.^105^ In the USA, a 2018 Associated Press National Opinion Research Center (AP–NORC) poll showed that 71% of Americans favored HHGE to prevent a child from being born with an incurable, potentially fatal disease.^106^ More recently, in 2020, a 20-country Pew Research Center study reported a global median of 60% support for using HHGE to reduce a baby’s risk of developing a serious disease over their lifetime, but only 14% support for using it to enhance traits such as intelligence.^107^

Public opinion in the Global South, where it has been measured, often reflects even greater receptiveness to HHGE. In India, for example, 56% of respondents in a 2020 Pew survey judged scientific research into gene editing to be ‘appropriate’—the highest of any country surveyed. In the same study, a majority of Indians expressed support for enhancement applications such as increasing a baby’s intelligence, in stark contrast to countries like Japan, where 86% of respondents rejected enhancement as a misuse of technology. This divergence illustrates that cultural context and local health priorities shape public attitudes as much as universal ethical concerns.

In summary, South Africans—when given the opportunity to deliberate—express strong support for HHGE, once safe and effective, to address pressing health challenges like TB and HIV, but reject enhancement uses. Globally, publics tend to follow the same broad pattern of high support for disease-related applications and low support for enhancement, with the South African case standing out for the intensity of support for disease immunity applications, reflecting the country’s unique health landscape.

### II.E. Synopsis

South Africa’s legal, social, and constitutional context points not toward prohibition but toward the regulated permissibility of HHGE. The NHA does not outlaw HHGE: section 57(1) proscribes manipulation of genetic material only when undertaken for reproductive cloning, while section 57(4) expressly authorizes research on zygotes up to 14 days with ministerial approval.^108^ Far from imposing a moratorium, the statutory framework anticipates regulated embryo research and leaves space for future governance of HHGE within an ethical and legal structure.

The country’s health landscape strengthens the rationale for such regulation. Unlike Europe or North America, South Africa continues to grapple with the dual epidemics of TB and HIV/AIDS alongside a rising burden of non-communicable diseases and significant rates of maternal, infant, and congenital disorders.^109^ In this setting, the appeal of novel biomedical interventions is sharper: a society that has pioneered large-scale antiretroviral rollout and TB diagnostics has also shown willingness to explore cutting-edge technologies.^110^ The possibility that HHGE could one day contribute to immunity against endemic diseases or prevention of inherited disorders must therefore be situated against these pressing public health realities rather than against abstract ethical concerns.

At present, the most relevant constitutional guarantee is the freedom of scientific research under section 16(1)(d).^111^ This right, though underdeveloped in South African jurisprudence, secures constitutional space for pioneering and controversial forms of inquiry, including HHGE. It protects the autonomy of scientists to pursue knowledge in contested domains, subject only to proportionate regulation necessary to safeguard other constitutional rights such as dignity and bodily integrity. Research into HHGE is therefore not only permissible but constitutionally protected, provided it is conducted within an ethical and regulated environment. Looking ahead, once HHGE matures into a demonstrably safe and effective technology, other rights will assume greater prominence. Jurisprudence such as *Treatment Action Campaign* establishes that the state has both a negative duty not to obstruct access to available healthcare and a positive duty to progressively realize equitable access,^112^ while *Surrogacy Advisory Group* confirms that reproductive technologies may not be prohibited on the basis of contested moral objections.^113^ In that future scenario, prospective parents would be constitutionally entitled to access HHGE as part of reproductive health care, free from moralistic restrictions and with a corresponding state duty to ensure fair availability.

Public opinion aligns with this trajectory. Deliberative engagement exercises show strong support for HHGE in preventing serious inherited conditions and, distinctively, in addressing TB and HIV while rejecting enhancement uses and insisting on equitable access.^114^ These findings mirror global patterns of support for therapeutic applications,^115^ but the intensity of endorsement for disease-related uses reflects South Africa’s epidemiological profile.^116^

Taken together, law, health realities, constitutional commitments, and public attitudes converge on a consistent principle: HHGE should be regulated rather than banned. The background therefore provides a clear normative foundation for understanding the significance of the 2024 NHREC guidelines.

## III. ANALYZING THE NHREC 2024 GUIDELINES

The NHREC’s 2024 guidelines can be understood as the institutional expression of the principles outlined above.^117^ South African law does not impose a blanket prohibition on HHGE; the country’s health realities generate pressing reasons to explore innovative responses; the Constitution protects both scientific freedom and access to healthcare;^118^ and public deliberation reveals strong support for disease-related applications.^119^ Taken together, these elements point not to prohibition but to careful regulation. The NHREC’s 2024 guidelines translated that orientation into practice.^120^ They did not license immediate clinical use; rather, they established a framework of anticipatory governance—regulating research now while preparing the ground for potential clinical application if and when science demonstrates safety and efficacy. Substantively, the guidelines embedded safeguards including scientific and medical justification,^121^ independent HREC review,^122^ transparency duties,^123^ informed consent,^124^ compliance with the statutory 14-day rule,^125^ ministerial authorization under section 57(4),^126^ and long-term monitoring obligations.^127^ The subsections that follow examine how these provisions addressed the distinctive features of HHGE.

### III.A. Scientific and Medical Justification

A central provision of the 2024 NHREC guidelines was that any HHGE research had to rest on a clear and compelling scientific and medical rationale.^128^ By design, the guidelines confined permissible research to interventions aimed at preventing serious genetic disorders or promoting immunity against life-threatening diseases.^129^ In doing so, they explicitly excluded speculative or enhancement-driven applications. This orientation ensured that HHGE research was channeled toward health outcomes of unquestionable public importance.

This design choice was hardly arbitrary: it was consistent both with South Africa’s health realities and with expressed public priorities.^130^ As discussed above, the country continued to carry the world’s highest burdens of TB and HIV/AIDS—conditions unimaginable as leading causes of death in the global north, yet daily realities in South Africa. Against this backdrop, the deliberative public engagement study on HHGE revealed overwhelming support for the technology’s use in preventing severe genetic conditions and, uniquely, in conferring immunity against TB and HIV.^131^ The guidelines’ insistence on targeting serious diseases therefore reflected a careful alignment with both epidemiological need and considered public opinion.

From an ethical perspective, this requirement embodied the principle of beneficence, directing scientific effort to interventions most likely to alleviate suffering and extend life. It also operationalized the principle of justice, ensuring that limited scientific and financial resources were prioritized toward those most vulnerable to disease and least able to access alternatives.

### III.B. Transparency and Informed Consent

Transparency and informed consent were central to the 2024 NHREC guidelines.^132^ Researchers were required to ensure that both participants and stakeholders were fully informed about the goals, methods, and implications of the research. This included obtaining consent from all directly involved parties, such as prospective parents and individuals whose genetic material was used in the research. By embedding these requirements, the guidelines gave practical effect to the ethical principle of autonomy, ensuring that individuals retained control over decisions with profound consequences for their lives. In constitutional terms, the guidelines upheld the right to human dignity,^133^ affirming the agency and worth of all who participated in the research process.

What distinguished these provisions from ordinary human-subject research was the requirement that transparency extend beyond the immediate research participants and continue throughout the entire research process. Unlike standard protocols, which focus primarily on consent at the point of enrolment, the NHREC guidelines demanded that ‘stakeholders’ more broadly be kept apprised of the goals, methods, and potential implications of HHGE research.^134^ In principle, this acknowledged that genome editing has implications far beyond the immediate circle of participants, touching future generations and society at large.

The difficulty, however, lay in execution. The guidelines did not define who counted as a stakeholder, nor did they provide criteria for research ethics committees to apply.^135^ In practice, this left HRECs to decide whether stakeholders should include professional societies such as the South African Society for Human Genetics, patient advocacy groups, relevant health authorities, research institutions themselves, or even the public at large. One could reasonably argue that the stakes of HHGE require at least some form of wider public engagement; yet the guidelines imposed no mechanism for ensuring this. The result was a framework that signaled openness but left its scope indeterminate.

By imposing a broader duty of openness without defining its content, the NHREC guidelines created both an ethical strength and a procedural weakness. On the one hand, they sought to secure accountability, build trust, and pre-empt the secrecy that has fueled global controversies. On the other hand, their vagueness risked inconsistent application and allowed critics to portray them as insufficiently inclusive. A clearer specification of who qualifies as a stakeholder—and whether public engagement is a requirement or an option—would have strengthened this provision considerably.

### III.C. Stringent Ethical Oversight

The guidelines required HHGE research to undergo rigorous review by health research ethics committees (HRECs).^136^ From the perspective of an HREC, participant protection was paramount: oversight was intended to minimize risks, secure informed consent, and safeguard the dignity and safety of all involved. This reflected the principle of non-maleficence, which obligates avoidance of harm.^137^ Yet the guidelines also directed HRECs to weigh principles of beneficence and justice. Committees were tasked with ensuring that research had the potential to deliver meaningful health benefits and to advance public health while distributing risks and benefits fairly.

Importantly, the guidelines embedded constitutional standards within this process. Ethical review was to be informed not only by generic bioethical principles but also by rights such as freedom of scientific research, access to healthcare, and human dignity. In this way, the guidelines rooted ethical oversight within South Africa’s constitutional order rather than external moralism. The weakness, however, was institutional: HRECs may not be fully equipped to adjudicate constitutional questions or long-term social implications. Without broader deliberative mechanisms, there was a risk that ethical review would narrow to procedural compliance rather than substantive constitutional reasoning.

### III.D. Ongoing Ethical Evaluation and Adaptation

The guidelines recognized that the ethical dimensions of HHGE could not be frozen in time.^138^ They therefore required researchers to re-evaluate their work continually as scientific knowledge advanced and new risks emerged. This commitment to responsiveness ensured that HHGE research would evolve in step with changing scientific, ethical, and legal standards. In constitutional terms, it echoed the ‘Treatment Action Campaign’ judgment,^139^ which held that health policy must be flexible, evidence-responsive, and open to adaptation. By mandating adaptability, the guidelines anticipated rather than ignored the uncertainties inherent in pioneering science.

### III.E. Safety and Efficacy

Safety and efficacy were non-negotiable requirements under the guidelines. Researchers were obligated to conduct thorough risk assessments and demonstrate that their methods met the highest scientific standards before proceeding.^140^ This dual emphasis on minimizing harm and demonstrating benefit reflected the principles of non-maleficence and beneficence.^141^

The difficulty, however, lies in calibration. The guidelines did not articulate what level of safety or efficacy would be sufficient in the distinctive context of HHGE, nor did they provide reference standards for ethics committees to apply. Would sufficiency require near-perfect precision, with almost zero risk of off-target effects, given that heritable alterations are passed to future generations? Or would an intervention be considered sufficiently safe if its foreseeable harms were less severe than the disease it was designed to prevent? These are radically different thresholds, yet the guidelines offered no guidance on how to navigate them.

Because HHGE raises unique, intergenerational risks, the absence of definitional clarity left too much to the discretion of individual research ethics committees, risking inconsistent judgments. Clearer guidance would have been valuable—both to ensure consistency across review bodies and to provide reassurance that decisions about safety and efficacy in this domain would not be left to ad hoc or subjective determinations.

### III.F. Long-Term Monitoring

The guidelines went further than immediate risk management, requiring researchers to commit to long-term monitoring of any individuals born following HHGE interventions.^142^ Such a provision is unusual in human-subject research, where proactive oversight typically ends once a trial concludes or after a limited follow-up period. In the case of HHGE, however, the subject is a future child whose permanently altered genome raises questions that extend far beyond conventional research horizons. Recognizing this singularity, the guidelines imposed obligations that accompany the individual across his or her lifetime.

By mandating extended follow-up, the guidelines served a dual purpose: protecting the ongoing health and well-being of those directly affected and generating essential knowledge about the long-term safety and efficacy of HHGE. Ethically, this requirement embodied beneficence—concern for the continuing welfare of individuals^143^—and non-maleficence, by systematically tracking and mitigating adverse effects that might emerge only over time.^144^

### III.G. Legal Compliance

The guidelines explicitly required compliance with all relevant South African laws, in particular the statutory 14-day rule for embryo research and the need for ministerial authorization under the NHA.^145^ This provision correctly reflected that section 57(1) of the NHA prohibits only reproductive cloning rather than genetic interventions per se, thereby affirming the existence of a lawful pathway for HHGE research.^146^ By requiring ministerial approval, the guidelines signaled an effort to anchor governance in democratic accountability.

At the same time, however, the provision underscored a key weakness. The guidelines merely reiterated the requirement of ministerial authorization but offered no criteria by which research ethics committees or prospective researchers could know what would count as an acceptable application, nor any standards against which the Minister would be expected to decide.^147^ In practice, this left both researchers and regulators in a position of uncertainty: investigators do not know what documentation or justification would satisfy the requirement, and the Minister retains unfettered discretion in granting or withholding approval. Such reliance on executive judgment introduced political fragility, since a single ministerial veto could halt HHGE research irrespective of constitutional guarantees of scientific freedom.

Had the guidelines provided objective criteria for ministerial approval—for instance, requiring demonstrable scientific merit,^148^ proportionality of risks and benefits,^149^ or alignment with constitutional principles such as dignity^150^ and equality^151^—they would have created a clearer and more reliable pathway. Without such benchmarks, the provision risks leaving HHGE research vulnerable not only to shifting political winds but also to inconsistent or opaque decision-making, undermining the very predictability and stability that anticipatory governance is meant to secure.

### III.H. Overall Assessment

Taken together, the NHREC’s 2024 guidelines offered a concise but substantively robust framework for HHGE research in South Africa. Each provision integrated core ethical principles while balancing constitutional rights, including dignity,^152^ bodily integrity,^153^ scientific freedom,^154^ and access to healthcare.^155^ In this respect, the guidelines marked a principled departure from moratorium approaches elsewhere, positioning South Africa as a jurisdiction willing to regulate rather than prohibit. Their central achievement was to demonstrate that scientific progress could be pursued without abandoning constitutional and ethical commitments.

Yet the framework’s brevity left important issues underdeveloped. Safety and efficacy were rightly treated as non-negotiable,^156^ but the absence of guidance on what thresholds should count as ‘sufficient’ left research ethics committees without clear standards in a uniquely high-stakes context. The provisions on transparency and stakeholder engagement recognized the broader social implications of HHGE but left ‘stakeholder’ undefined,^157^ creating uncertainty about whether this category should include the public at large, patient groups, or only professional bodies. Reliance on ministerial authorization under s 57(4) provided a lawful pathway but exposed the framework to political discretion,^158^ while the silence on how equal access might be secured sat uneasily alongside South Africa’s constitutional commitment to healthcare rights.^159^ These were real areas where the framework could have benefited from further detail.

However, these weaknesses were overlooked in the public controversy that followed.^160^ As we explore in the next section, critics did not engage with the guidelines on their merits; rather, they attacked them simply because they contemplated the possibility of children born following HHGE interventions. The problem with this critique was that anticipation was conflated with permission. The guidelines did not offer an unqualified green light to clinical application but instead set out a sequence of ethical and legal thresholds that would need to be satisfied before any such application could be contemplated. Clinical use is not scientifically feasible at present, as HHGE remains neither efficient nor safe enough for use in humans—even if these concepts are given broad meanings. The guidelines were therefore not an instrument of immediate authorization, but of anticipatory governance—ensuring that, should science advance, South Africa would be prepared with an ethical and legal framework already in place. Far from being naïve, we suggest that this forward-looking orientation reflected a responsible effort to balance innovation with constitutional safeguards and public accountability.

## IV. ACADEMIC CRITIQUE OF THE NHREC’S 2024 GUIDELINES ON HHGE

The NHREC’s 2024 guidelines on HHGE became a focal point in debates over law and science governance. Among the most prominent critiques were those by Françoise Baylis, Katie Hasson, Jantina de Vries, and Michè​le Ramsay.^161^ These interventions illustrate central dynamics of bioscience politics: the elevation of bioethics-elite perspectives over public opinion, and the mobilization of statutory interpretation as a political tool. Their critiques of the NHREC’s 2024 guidelines must therefore be read not only as disagreements over doctrinal meaning, but as part of a broader contest over whose voices and values are authorized to define the permissible trajectory of genome editing. The following subsections examine their interventions, in turn.

### IV.A. Baylis and Hasson

Françoise Baylis and Katie Hasson’s intervention on South Africa’s 2024 NHREC guidelines must be situated within the broader politics of global bioethics.^162^ Baylis has long been a prominent voice advocating caution in the governance of HHGE, most notably as a member of the WHO Expert Advisory Committee on Developing Global Standards for the Governance and Oversight of Human Genome Editing (2019–21).^163^ In that capacity, she consistently argued for a restrictive, precautionary global framework.^164^ Hasson, in turn, is affiliated with the Center for Genetics and Society, an organization publicly committed to opposing HHGE on ideological grounds.^165^ Their joint intervention in the South African debate thus reflects positions shaped less by South Africa’s legal or social context than by a shared global orientation toward restraint and scepticism.

Their critique of the NHREC guidelines advanced two central claims: first, that the guidelines enabled ‘genetically modified babies’ without adequate safeguards;^166^ and second, that they were inconsistent with section 57(1) of the NHA.^167^ Both claims are doctrinally and empirically unsustainable. As demonstrated in the constitutional analysis above, the South African framework protects freedom of scientific research and the right of access to healthcare.^168^ The NHREC guidelines sought precisely to operationalize these guarantees: they confined permissible HHGE research to serious medical rationales,^169^ required ministerial authorization,^170^ enforced the statutory 14-day limit on embryo research,^171^ and mandated oversight,^172^ informed consent,^173^ and long-term monitoring.^174^ Thus, the guidelines embedded multiple safeguards, aligning with constitutional principles as well as with the ethical norms of beneficence, non-maleficence, autonomy, and justice.

The legal critique is particularly problematic. In their 2020 global policy landscape survey, Baylis, Hasson, and their South African collaborator Jantina De Vries explicitly reported that South Africa had no laws relevant to HHGE^175^—despite the fact that the NHA was enacted in 2003.^176^ The subsequent claim, in 2024, that section 57(1) of the NHA prohibits HHGE thus marks a dramatic reversal without explanation. As demonstrated in the statutory analysis above, the clause prohibits only genetic manipulation ‘for the purpose of reproductive cloning’.^177^ Collapsing this into a blanket prohibition disregards the statutory text and erases the categorical distinction between cloning and genome editing. By contrast, the NHREC guidelines explicitly required legal compliance, thereby respecting both the letter of the law and the rule of law itself.

Equally significant is their silence on public opinion. Their critique made no reference to the South African deliberative engagement study,^178^ which revealed broad conditional support for HHGE in health contexts and no principled opposition among participants. The strongest support arose in relation to immunity against TB and HIV, diseases that shape South Africa’s epidemiological landscape.^179^ To ignore these findings, while depicting the guidelines as reckless, reproduces a familiar biopolitical pattern: Global North commentators present their restrictive frameworks as universal, while local democratic deliberation is disregarded.

Seen in this light, Baylis and Hasson’s intervention exemplifies how international actors use ethical rhetoric and incorrect statutory interpretation to shape domestic governance debates. Their critique overstates legal prohibitions, under-represents embedded safeguards, and sidelines empirical public opinion. As analyzed in earlier sections, South Africa’s constitutional and statutory framework permits a more nuanced reading—one that affirms both scientific freedom and equitable access to health innovations.^180^ The Baylis and Hasson intervention thus illustrates the dynamics of bioscience politics: whose interpretations are elevated as authoritative and whose are discounted. In this respect, their position—rooted in Baylis’s WHO committee role and Hasson’s civil society advocacy—reflects an ideological project of precaution that inadequately engages with the constitutional, social, and public health realities of South Africa.^181^

### IV.B. De Vries

Jantina de Vries’s role in the South African debate must be situated within the broader terrain of bioscience politics. She was the South African collaborator for Baylis et al.’s 2020 global survey of HHGE governance,^182^ which at the time concluded that no relevant South African legislation addressed HHGE. Subsequently, she was appointed to the WHO Expert Advisory Committee on Human Genome Editing.^183^ Yet prior to 2024, she had not published peer-reviewed work on HHGE’s legal or ethical dimensions. Her entry into this discourse was thus mediated less by a sustained record of scholarship than by the authority conferred through international governance networks. This trajectory illustrates how epistemic authority in HHGE is often produced transnationally: through institutional affiliations and committee participation that precede substantive engagement with domestic legal frameworks.

In a November 2024 blog post on the University of Cape Town’s EthicsLab site, De Vries advanced three main claims: first, that our legal interpretation^184^ of section 57(1) is ‘highly technical’ and unconvincing; second, that ‘most legal and scientific experts’ consider HHGE unlawful under current law; and third, that our work forms part of a broader ‘strategy’ toward litigation.^185^ The framing of our research as a ‘minority view’, as ‘controversial’, and as embedded in a ‘strategy’ is significant. Such descriptors function not merely as neutral characterizations but as rhetorical devices that position our scholarship as marginal, even suspect, and thereby reduce its standing in the broader academic and policy debate. While disagreement is inherent in scholarly exchange, the reliance on these delegitimizing tropes displaces substantive engagement with the legal argument itself.

Doctrinally, her reading of section 57(1) mirrors that of Baylis and Hasson: she collapses the statutory text into a categorical prohibition. As established in the statutory analysis above, this disregards the controlling qualifier ‘for the purpose of reproductive cloning’.^186^ Like Baylis and Hasson, ^187^ her invocation of the NHA represents a marked reversal from the 2020 position (to which she herself contributed as a country collaborator) that South Africa had no relevant HHGE law. No acknowledgment is made of this reversal, nor of the interpretive reasoning that would explain such a shift.

Her comments on our deliberative public engagement study likewise adopt a dismissive register. She refers only to unspecified ‘methodological concerns’ and asserts that the findings overstate public support. Yet, as demonstrated in Section II.D, the study revealed broad conditional support for HHGE in health contexts, with access and equity emerging as central themes, and no principled opposition among participants.^188^ To dismiss this evidence without a detailed critique undercuts the value of deliberative democratic engagement and reflects a recurring biopolitical pattern in which local public reasoning is subordinated to elite caution.

Seen in context, De Vries’s intervention reproduces the same set of moves observed in Baylis and Hasson’s critique: overstate legal prohibitions, understate safeguards, discount empirical evidence of public opinion, and characterize South African legal scholarship as aberrant or strategically motivated. Each of these moves aligns more closely with the reproduction of global governance orthodoxies than with a context-sensitive engagement with South Africa’s constitutional framework,^189^ statutory text,^190^ and public deliberation.^191^

### IV.C. Ramsay et al.

Ramsay et al.’s 2024 editorial in the *South African Medical Journal* styles itself as a ‘reality check’ on claims that HHGE is lawful in South Africa.^192^ Despite passing reference to the NHREC 2024 guidelines, the substance of the piece is directed at our research group’s well-known interpretation of section 57(1) of the NHA.^193^ The headline functions as an admonition: an assertion of authoritative correction of our legal analysis rather than an invitation to collegial debate.

This intervention must also be situated within the global governance networks already discussed. The first author, Ramsay, is a distinguished geneticist and former member of the US/UK-led *International Commission on the Clinical Use of Human Germline Genome Editing*;^194^ her co-author De Vries served on the WHO Expert Advisory Committee.^195^ Those affiliations confer epistemic authority in transnational governance, but they do not, by themselves, secure doctrinal accuracy in the interpretation of South African law.

The editorial’s central legal claim is that HHGE is prohibited by section 57(1) of the NHA.^196^ Yet their presentation of the statutory text is inaccurate. In their version, the crucial qualifying phrase—‘for the purpose of the reproductive cloning of a human being’^197^—is treated as if it attaches only to subsection (b). In the statute, however, the qualifier is set off from the subsections and applies equally to (a) and (b). By omitting the line break and indentation, the editorial collapses what is, in law, a narrow prohibition on reproductive cloning into a blanket ban on all manipulation of genetic material. This is not a trivial editorial slip: it materially alters the meaning of the provision and risks misleading readers unfamiliar with the statute. At minimum, it reflects a failure to appreciate the interpretive significance of statutory structure.

Moreover, reading the qualifier as attaching only to subsection (b) would generate untenable consequences. It would criminalize not only HHGE but also routine manipulation of genetic material, including DNA isolation, sequencing, and IVF. Yet IVF is explicitly regulated in subsidiary legislation,^198^ which presupposes its legality; this legislative context strongly militates against the interpretation advanced by Ramsay et al.^199^ That Ramsay et al. did not recognize these ramifications underscores the superficiality of their statutory engagement: their interpretation collapses the regulatory framework into incoherence without their appearing even to notice.

In our published response,^200^ we drew attention to both the misquotation of section 57(1) and the untenable consequences that followed from it. Rather than engaging substantively, Ramsay et al. ^201^ in their reply sought to downplay the issue, asserting that statutory formatting ‘does not determine legality’, and retreating instead to legislative ‘intent’.^202^ They further escalated the rhetoric by characterizing our interpretation as ‘ill-informed and dangerous’.^203^ Yet interpretive fidelity in South African law indeed requires attention to statutory formatting: ignoring it in a way that changes the provision’s meaning cannot be excused by unsubstantiated appeals to intent. Had Ramsay et al. acknowledged their initial error and the doctrinal incoherence it produced, the debate might have advanced constructively. Instead, their reply entrenches a misreading that collapses cloning and editing into a single category, substituting accusation for careful statutory analysis.

Their invocation of intent is, in any event, unpersuasive. They imply that Parliament’s purpose in enacting section 57(1) was to outlaw HHGE but provide no reasons for this assumption. On the contrary, a contextual reading strongly suggests otherwise. Enacted in 2003, in the aftermath of Dolly the Sheep,^204^ the provision reflects contemporaneous global anxieties about reproductive cloning—not genome editing, which at the time was neither technically advanced nor ethically salient. The section heading (‘Prohibition of reproductive cloning of human beings’),^205^ repeated references to cloning, and definitional clauses all confirm this narrow mischief. Extending section 57(1) into a blanket prohibition of HHGE imports a legislative purpose for which there is no historical or contextual support.

Thus, the appeal to ‘intent’ is unsubstantiated, and the refusal to correct a material misquotation undermines the integrity of their intervention. On a faithful reading of the text, structure, and context of sections 57(1) of the NHA, it proscribes reproductive cloning and does not enact a categorical ban on HHGE.

Beyond statutory interpretation, the editorial adopts a dismissive tone toward existing South African scholarship. Ramsay et al. criticize our group for relying largely on our own publications, presenting this as a lack of scholarly rigor.^206^ Yet this charge ignores the reality that ours is, to date, the only research group that has produced sustained, peer-reviewed work on HHGE in South Africa. In the absence of alternative domestic scholarship, self-citation is not a strategy of self-legitimation but a reflection of the field’s actual state of knowledge. By contrast, none of the authors of Ramsay et al. has published prior peer-reviewed legal or ethical analysis of HHGE in South Africa; their claim to authority rests primarily on committee participation at the WHO and other international bodies.

Their treatment of public engagement further illustrates a biopolitical script. Ramsay et al. criticize the South African deliberative public engagement study for inviting participants to consider hypothetical safe and effective scenarios,^207^ implying that this undermines its validity. Yet as shown in Section II.D, the study revealed broad conditional support for HHGE in therapeutic contexts and opposition to enhancement uses.^208^ To dismiss these findings as methodologically flawed without engaging the study design or published results is to reproduce a familiar pattern: marginalizing Global South public reasoning in favor of elite governance discourses.

Finally, the framing of their editorial—cast as a ‘reality check’^209^—positions their perspective as authoritative and others’ as naïve or misguided. This rhetorical stance echoes Baylis and Hasson’s ‘ethical precipice’ imagery,^210^ locating South Africa at risk of recklessness while overlooking the constitutional safeguards of freedom of scientific research and access to healthcare (Sections II.B and II.C).^211^ It also exemplifies how bioscience politics operate: international experts leverage governance prestige to discipline domestic debates, often through rhetorical delegitimation rather than doctrinal engagement.

Taken together, Ramsay et al.’s^212^ intervention illustrates the dynamics of global biopolitics at work in the South African case. Their misrepresentation of section 57(1),^213^ their dismissal of the only sustained domestic scholarship, and their marginalization of empirical public engagement data all function to foreclose local agency while elevating transnational governance orthodoxies. As with Baylis, Hasson, and De Vries, the result is a dramatic about-face from the earlier 2020 claim that South Africa had no relevant HHGE law, now reinterpreted—through misquotation—as a categorical prohibition. The South African debate, however, cannot be advanced by rhetorical ‘reality checks’^214^ that obscure rather than illuminate the actual constitutional, statutory, and social realities of HHGE governance.

### IV.D. Synopsis

Taken together, the interventions by Baylis and Hasson,^215^ De Vries,^216^ and Ramsay et al.^217^ illustrate the dynamics of bioscience politics in the governance of HHGE in South Africa. Each critique shared three notable shortcomings. First, none engaged substantively with the safeguards and regulatory mechanisms embedded in the NHREC’s 2024 guidelines,^218^ preferring instead to characterize the framework as reckless. Second, all relied on a misrepresentation of section 57(1) of the NHA,^219^ transforming a narrow prohibition on reproductive cloning into a political tool for portraying HHGE as categorically unlawful. Third, they either ignored or dismissed South African public opinion, despite evidence from deliberative engagement showing broad conditional support for HHGE in therapeutic contexts and no principled opposition. These moves—silencing local public reasoning, overstating legal prohibitions, and disregarding actual regulatory safeguards—suggest that the critiques were driven less by doctrinal substance than by ideological opposition. The result was a discursive strategy that sought to delegitimize the NHREC’s 2024 guidelines on HHGE and those who supported it, rather than to engage constructively within South Africa’s constitutional, statutory, and social context.

## V. INSTITUTIONAL RESPONSES AND THE REPEAL OF THE 2024 NHREC GUIDELINES

The academic critiques of the NHREC’s 2024 guidelines on HHGE were quickly taken up and amplified by institutions. Recall, however, that the process by which the NHREC developed these guidelines was itself narrowly framed. Consultation prior to their adoption was confined to health research ethics committees formally registered with the NHREC—that is, the established network of ethics reviewers.^220^ Absent from this process were geneticists and their professional society; research groups working specifically on the ethical, legal, and social implications of HHGE; and the broader public. As a result, the guidelines, although substantively robust, lacked the participatory legitimacy that broader consultation might have provided. This structural fragility helps explain why, once academic critiques emerged, institutional actors found it easier to adopt and amplify them rather than defend the guidelines on their own terms.

### V.A. NHREC Statement

The NHREC’s 2024 guidelines explicitly recognized the promise of HHGE, stating: ‘Research on heritable human genome editing (HHGE) holds significant potential for addressing genetic diseases and improving human health’.^221^ The very act of establishing an ethical framework presupposed lawfulness: regulating what is categorically prohibited would be incoherent.

Yet in November 2024, amid mounting criticism, the Chairperson of the NHREC issued a public statement asserting that HHGE is unlawful under South African law.^222^ This claim relied on section 57(1)—which prohibits reproductive cloning—and section 57(4)—which allows ministerial authorization of embryo research under 14 days.^223^ As shown in our statutory analysis above, the reliance on section 57(1) is misplaced: HHGE is distinct from reproductive cloning, and the provision’s controlling qualifier makes this clear. Moreover, section 57(4) provides a direct legal pathway for HHGE research with ministerial approval.

The statement thus reflects not a coherent legal position but a hurried attempt to placate alarmist critiques that had painted South Africa as teetering on an ‘ethical precipice’.^224^ This episode foreshadowed the Council’s eventual retreat from its own guidelines and raised questions about its credibility as a regulatory body.

### V.B. South African Society of Human Genetics

The following month, the South African Society of Human Genetics convened an ‘indaba’ (discussion forum) on HHGE.^225^ The panel comprised five experts, four of whom—including Michèle Ramsay and Jantina de Vries—would soon co-author the Ramsay et al. editorial. No proponents of the NHREC’s 2024 guidelines were invited to serve on the expert panel.

Predictably, the consensus statement that followed moved in only one ideological direction: it endorsed a moratorium on HHGE and criticized the guidelines for ‘ambiguity’,^226^ though without identifying what, precisely, was ambiguous. Once again, section 57(1) was invoked as prohibiting HHGE but without any sustained interpretation of the statutory text.

This exclusionary framing ensured that no corrective engagement was possible. By excluding voices supportive of the NHREC’s guidelines, the ‘indaba’ foreclosed opportunities to address misunderstandings of either the statutory framework or the guidelines themselves. What emerged was less a genuine consensus than a manufactured one—produced through the closed echo chamber of like-minded experts and the uncritical repetition of a mischaracterized legal claim.

### V.C. Repeal of the Guidelines

In the first half of 2025, the NHREC repealed the 2024 HHGE guidelines altogether. In their place, a placeholder paragraph was inserted:

Given the ongoing debates on heritable human genome editing research, there is a need for further national stakeholder engagement to guide the update of ethics review in this area of research. These updates will be communicated to RECs [research ethics committees] accordingly.^227^

This repeal marked the culmination of a process of less than a year in which statutory misinterpretation was elevated into institutional orthodoxy. The result was not an amended or clarified framework but the wholesale abandonment of regulation. Safeguards unique to the 2024 guidelines—such as strict medical-need justification,^228^ enhanced oversight requirements,^229^ and obligations for long-term monitoring^230^—were simply abandoned, replaced by a vague commitment to indefinite future dialogue.

The placeholder paragraph’s reference to the need for ‘further national stakeholder engagement’ is telling.^231^ Having initially limited its consultations to research ethics committees, the NHREC now tacitly acknowledged that a much broader deliberative process is indispensable. Yet without clarity on whether such engagement will be genuinely inclusive, and without correcting the statutory misinterpretations that have already been institutionalized, there is a risk that this recognition remains symbolic.

## VI. CONCLUSION

The South African controversy over HHGE illustrates how law, ethics, and politics intertwine in shaping the governance of emerging technologies. The repeal of the NHREC’s 2024 guidelines was not the product of substantive legal analysis or empirical ethical deliberation but of a cascade of academic critiques and institutional responses that reinforced one another without adequate engagement with either the statutory framework or the safeguards the guidelines contained. From Baylis and Hasson’s alarmist framing,^232^ through De Vries’s dismissive interventions,^233^ to Ramsay et al.’s misquotation of section 57(1),^234^ a common thread runs: critiques have been more rhetorical than analytical, resting on mischaracterizations of the law and elisions of the guidelines’ carefully designed protections.

Institutional actors, rather than correcting these misrepresentations, amplified them. The NHREC chair’s November 2024 statement that HHGE is ‘unlawful’ exemplified the tendency to conflate cloning and genome editing under section 57(1), notwithstanding the statutory qualifier and the explicit legal pathway for embryo research under section 57(4). The South African Society of Human Genetics’ ‘indaba’ the following month compounded this dynamic: by excluding any proponents of the guidelines, it foreclosed substantive debate and issued a consensus statement endorsing a moratorium.^235^ Its statement once again repeated the claim that section 57(1) prohibits HHGE, without offering any sustained interpretation of the statutory text. This repetition illustrates how misreadings, once articulated by authoritative figures, can replicate across forums without reflection or correction. By mid-2025, the NHREC itself capitulated to this pressure, repealing the guidelines and replacing them with a placeholder paragraph that deferred regulation indefinitely.^236^

While the 2024 guidelines were substantively strong—a framework aligned with constitutional rights and embedding safeguards such as ministerial authorization,^237^ compelling medical justification,^238^ informed consent,^239^ and long-term monitoring^240^—they also displayed weaknesses. Concepts central to governance, such as what should count as ‘sufficient’ safety and efficacy, or who qualifies as a stakeholder for purposes of transparency, were left undefined. Reliance on ministerial discretion under section 57(4)^241^ without objective criteria also left scope for inconsistency. Yet these substantive lacunae were not the basis of the criticisms that ultimately sank the guidelines. Instead, they became collateral damage in a debate framed almost exclusively in terms of political optics. Procedurally, moreover, the NHREC compounded fragility by consulting only registered research ethics committees prior to adoption. This narrow process left the guidelines without broad participatory legitimacy, a weakness later underscored by the placeholder paragraph’s call for ‘further national stakeholder engagement’.^242^ What followed was not refinement of the framework but its collapse into a regulatory vacuum driven more by rhetorical alarm than reasoned deliberation.

These developments underscore several broader themes of bioscience governance. First, the process of developing ethical and legal instruments matters: without broad and inclusive consultation, even robust instruments are vulnerable. Second, the law itself became a political instrument. Instead of serving as a framework for constructive ethical dialogue, statutory interpretation was wielded as a rhetorical device: the claim that section 57(1)^243^ prohibited HHGE hardened into authoritative orthodoxy, foreclosing debate. Its logical consequences—if taken seriously—would extend far beyond HHGE, criminalizing routine and widely accepted practices such as DNA isolation, sequencing, and even IVF. That such implications went unacknowledged^244^ only underscores the absence of sustained legal engagement in the debate. Third, elite academic opinion prevailed over democratic input. South African public engagement research had shown conditional but substantial support for HHGE in health contexts,^245^ yet this evidence was ignored, dismissed, or marginalized. Fourth, professional and institutional dynamics reinforced conformity. The adoption of a moratorium by the national genetics society, ^246^ while not legally binding, signaled to researchers what is deemed socially and professionally acceptable, adding informal pressure that reinforced the NHREC’s formal repeal of the guidelines.

The cumulative effect is that statutory misinterpretations, once institutionalized, risk chilling both ethical debate and scientific exploration. It is difficult to deliberate meaningfully on the conditions under which HHGE might be pursued if the starting assumption, however unfounded, is that the practice is categorically unlawful. Before the NHREC’s promised stakeholder engagement can fulfill its potential, there must be a shared acknowledgment of the actual legal position: that the NHA prohibits reproductive cloning but does not enact a blanket ban on HHGE. Without this recognition, dialogue risks proceeding under a misapprehension that precludes constructive ethical or policy debate.

At stake is not merely the accuracy of statutory interpretation but the trajectory of governance itself. Refusing to envision the future of science is itself a regulatory choice—one that risks leaving a governance vacuum should scientific advance arrive more rapidly than anticipated. The NHREC’s 2024 guidelines represented a future-ready regulatory architecture: they acknowledged both the present limits of science and the importance of preparing responsibly and transparently for what might lie ahead.^247^ The failure of critics to appreciate this distinction led them to misrepresent the guidelines as reckless,^248^ when in fact their most forward-looking feature was their willingness to plan for the possibility of responsibly governed clinical application.

Looking forward, the South African experience offers a cautionary lesson for global governance. It shows that substantive strength alone is insufficient: even a framework that is substantively sound, ethically rigorous, and legally coherent can collapse if it is not grounded in a robust procedural foundation. The 2024 guidelines were attacked and ultimately withdrawn, not because of their genuine substantive shortcomings but because they dared to contemplate a regulated pathway to clinical HHGE. Critics bypassed the finer points of their weaknesses and instead mobilized alarm at the prospect of genetically modified babies,^249^ even though the rhetoric of ‘designer babies’ was found to be confounding in the opinion of Justice Khampepe in the *AB and Another v. Minister of Social Development* case.^250^ What proved decisive was not the content of the framework, but the absence of broad-based consultation to lend it political legitimacy. Substantive rigor without procedural legitimacy left the guidelines exposed to rhetorical assault and political retreat.

The episode also illustrates how ethical deliberation can be distorted when statutory interpretation is deployed as a political instrument, how international prestige can overshadow local constitutional guarantees, and how easily publics most affected can be sidelined. Yet it also points to a possible path forward. A genuinely inclusive stakeholder process—attentive to constitutional rights, to empirical public opinion, and to rigorous statutory interpretation—could re-establish the conditions for balanced debate. The NHREC has now committed itself to further stakeholder engagement; the question is whether it will deliver a process that is genuinely broad-based and deliberative, or allow the placeholder paragraph to persist. If South Africa is to contribute meaningfully to global governance of HHGE, its legal and ethical debates must proceed with fidelity to the text and context of the law, in a procedurally inclusive manner that takes due account of public opinion, and always grounded in the constitutional values of freedom, dignity, and equality.^251^

## Supplementary Material

lsaf029_Statement_NHREC_heritable_human_genome_editing_November_2024

## Footnotes

1 For a discussion on the transformative potential of HHGE for preventing genetic diseases, see eg, N. Yadav & B.K. Thelma, *Human Heritable Genome Editing-Potential and Current Status for Clinical Use*, 23 ABDR 1 (2021).; For the broader applications of genome editing technologies in combating infectious diseases, see eg, Y. Zhang & M. Li, *Genome Editing Technologies as Cellular Defense Against Viral Pathogens*, 9 Front. cell dev. Biol. 716344 (2021).

2 D. Thaldar, M. Botes, B. Shozi, B. Townsend & J. Kinderlerer, *Human Germline Editing: Legal-Ethical Guidelines for South Africa*, 116 S. Afr. J. Sci e6760 (2020).

3 D. Thaldar & B. Shozi, *Procreative Non-Maleficence: A South African Human Rights Perspective on Heritable Human Genome Editing*, 3 CRISPR J. 245 (2020).

4 T. Kamwendo, *Access to Healthcare in the Age of CRISPR: An Analysis of the Right to Heritable Human Genome Editing in the Context of the Tuberculosis Epidemic in South Africa* (PhD thesis, Univ. of KwaZulu-Natal 2022), https://researchspace.ukzn.ac.za/handle/10413/21540 (accessed Nov. 3, 2025).

5 D. Thaldar, *A Golden Opportunity for South Africa to Legislate on Human Heritable Genome Editing*, 16 S. Afr. J. Bioethics Law e1568 (2023).

6 L. Wiley, M. Cheek, E. LaFar et al., *The Ethics of Human Embryo Editing via CRISPR-Cas9 Technology: A Systematic Review of Ethical Arguments, Reasons, and Concerns*, 37 HEC F*.* 267 (2025).

7 A. Kwarteng, S.T. Ahuno & G. Kwakye-Nuako, *The Therapeutic Landscape of HIV-1 via Genome Editing*, 14 Aids Res. Ther. 32 (2017); Vivek Naranbhai, *The Role of Host Genetics (and Genomics) in Tuberculosis*, 4 Microbiol. Spectr. (2016).

8 A. V. Anzalone et al., *Search-and-Replace Genome Editing Without Double-Strand Breaks or Donor DNA*, 576 Nature 149 (2019).; A.C. Rossidis et al., *In Utero CRISPR-Mediated Therapeutic Editing of Metabolic Genes*, 24 Nat. Med. 1513 (2018).

9 Nat’l Health Research Ethics Council, *South African Ethics in Health Research Guidelines – Principles, Processes and Structures* (3d ed., version 1, Nat’l Dep’t of Health 2024) at 74–75. This first version of these Guidelines is no longer available on the Nat’l Dep’t of Health’s website. As such, it is provided as Supplementary Document 1.

10
*Id.*

11 F. Baylis & K. Hasson, *South Africa Amended Its Research Guidelines to Allow for Heritable Human Genome Editing*, The Conversation, https://theconversation.com/south-africa-amended-its-research-guidelines-to-allow-for-heritable-human-genome-editing-241136 (accessed Dec. 16, 2024); J. De Vries*, The Legal and Ethical Debate on Human Heritable Genome Editing (HHGE) in South Africa, Health Ethics Lab*, https://health.uct.ac.za/ethics-lab/articles/2024-11-15-legal-and-ethical-debate-human-heritable-genome-editing-hhge-south-africa (accessed Dec. 16, 2024); M. Ramsay, M. Pepper, J. De Vries, S. Mahomed, & E. Flack-Davison, *Heritable Human Genome Editing in South Africa – Time For a Reality Check*, 115 S. Afr. Med. J. e2872 (2025).

12 Constitution of the Republic of South Africa, 1996.

13 D. Thaldar, B. Shozi, M. Steytler, G. Hendry, M. Botes, N. Mnyandu et al., *A Deliberative Public Engagement Study on Heritable Human Genome Editing Among South Africans: Study Results*, 17 Plos One e0275372 (2022).

14 NHREC, *supra* note 9, § 4.3.2, at 74–75.

15 Kamwendo, *supra* note 4; Constitution, *supra* note 12.

16 NHREC, *supra* note 9, § 4.3.2, at 74–75.

17 Kamwendo, *supra* note 4.

18 D. Thaldar, *Clarifying Misconceptions and Recentring the Debate on Heritable Human Genome Editing in South Africa: A Response to De Vries*, 28 PER/PELJ (2025), http://dx.doi.org/10.17159/1727-3781/2025/v28i0a21163; Baylis & Hasson, *supra* note 10; De Vries, *supra* note 10; Ramsay et al., *supra* note 10.

19 National Health Act 61 of 2003 (S. Afr.).

20
*Id.* § 57(1).

21
*Minister of Health v. New Clicks S. Afr. (Pty) Ltd*, 2006 SA 311 (CC).

22 Merriam-Webster, Manipulate, Merriam-Webster.com  *Dictionary*, https://www.merriam  webster.com/dictionary/manipulate (accessed Dec. 7, 2024).

23 Cambridge University Press, *Manipulate*, Cambridge Academic Content Dictionary, https://dictionary.cambridge.org/dictionary/english/manipulate (accessed Dec. 7, 2024).

24 NHA, *supra* note 19, § 57(1).

25 See, eg, *Loughrin v. United States*, 573 U.S. 351 (2014), where the U.S. Supreme Court treated line breaks and indentation as relevant contextual indicators that statutory clauses were independent and equally operative.

26 National Academy of Sciences (US), National Academy of Engineering (US), Institute of Medicine (US) and National Research Council (US) Committee on Science, Engineering, and Public Policy, *Cloning: Definitions and Applications, in*  Scientific and Medical Aspects of Human Reproductive Cloning. (Nat’l Acads. Press (U.S.), 2002).

27 NHA, *supra* note 19, § 54(7).

28 Y. Xue & L. Shang, *Are We Ready for the Revision of the 14-Day Rule? Implications From Chinese Legislations Guiding Human Embryo and Embryoid Research*, 10 Front. Cell Dev. Biol. 1016988 (2022), https://doi.org/10.3389/fcell.2022.1016988.

29
*Id.*

30 Choice on Termination of Pregnancy Act 92 of 1996 (S. Afr.)

31 NHA, *supra* note 19, § 54(7).

32
*Id.* § 71.

33
*Id.* § 71(1).

34
*Id.* § 71(2).

35
*Id.* § 71(3).

36
*Id.* § 72.

37 The Regulations Relating to Research with Human Participants of 2014 (S. Afr.)

38 NHA, *supra* note 19, § 71.

39
*Id.* § 72.

40 Regulations Relating to Research with Human Participants, *supra* note 37.

41 World Health Org., *New Data: Noncommunicable Diseases Cause 1.8 Million Avoidable Deaths and Cost US$514 Billion Every Year, Reveals New WHO/Europe Report,*  https://www.who.int/europe/news/item/27-06-2025-new-data--noncommunicable-diseases-cause-1-8-million-avoidable-deaths-and-cost-us-514-billion-USD-every-year--reveals-new-who-europe-report (accessed Aug. 24, 2025); S.L. Murphy, K.D. Kochanek, J.Q. Xu & E. Arias, *Mortality in the United States, 2023*, NCHS Data Brief No. 521 (Nat’l Ctr. for Health Statistics 2024), https://dx.doi.org/10.15620/cdc/170564.

42 World Health Org., *Global Tuberculosis Report 2023*, https://www.who.int/teams/global-programme-on-tuberculosis-and-lung-health/tb-reports/global-tuberculosis-report-2023 (accessed Mar. 15, 2025).

43 United Nations Programme on HIV/AIDS, *Country Factsheet: South Africa 2023*, https://www.unaids.org/en/regionscountries/countries/southafrica (accessed Mar. 10, 2025).

44
*Id.*

45 Stats SA, *Mortality and Causes of Death in South Africa: Findings from Death Notification*, *2020*, Statistical Release, at 5 (2024).

46 WHO, *supra* note 42.

47 UNAIDS, *supra* note 43.

48 World Health Org., *Trends in Maternal Mortality: 2000 to 2020*, at 12 (WHO, UNICEF, UNFPA, World Bank Grp. & U.N. DESA/Population Div. 2023).

49 United Nations Inter-agency Group for Child Mortality Estimation, *Levels & Trends in Child Mortality 2022*, at 8 (2022).

50 Stats SA, *supra* note 45, at 9.

51 UNAIDS, *supra* note 43.

52 South African Med. Research Council, *Gene Therapy & Gene Editing Research in South Africa (2023)*, https://www.samrc.ac.za/news/gene-therapy-editing (accessed Mar. 10, 2025).

53 G. Sveinbjornsson et al., *HLA Class II Sequence Variants Influence Tuberculosis Risk in Populations of European Ancestry*, 48 Nat. Genet. 318 (2016).

54 B. Aksak-Wąs & M. Parczewski, *Genetic Factors Influencing HIV Infection: A Review*, 22 HIV AIDS Rev. (2022).

55 B. Qie, J. Tuo, F. Chen, H. Ding & L. Lyu, *Gene Therapy for Genetic Diseases: Challenges and Future Directions*, 6 medcomm (2020) e70091 (2025).

56 H.L. Malherbe, C. Aldous, A.L. Christianson, M.W. Darlison & B. Modell, *Modelled Epidemiological Data for Selected Congenital Disorders in South Africa*, 12 J. Community Genet. 357 (2021); S. Moosa, K.C. Coetzer, E. Lee & G.H. Seo, *Undiagnosed Disease Program in South Africa: Results From First 100 Exomes*, 188 Am. J. Med. Genet. A 2684 (2022).

57 M. Khumalo, *Porphyria*, 29 S. Afr. J. Anaesth. Analg. S54 (2023); J.G.R. Kromberg, P. Manga & R. Kerr, *Children With Oculocutaneous Albinism in Africa: Characteristics, Challenges and Medical Care*, 14 S. Afr. J. Child Health 50 (2020); A.D. Marais, D.J. Blom & F.J. Raal, *Familial Hypercholesterolaemia in South Africa: A Reminder*, 111 S. Afr. Med. J. 700 (2021).

58 Constitution, *supra* note 12, §§ 10, 27.

59
*Minister of Health v. Treatment Action Campaign* (No. 2), 2002 (5) SA 721 (CC) (S. Afr.).

60
*Id.*

61 Constitution, *supra* note 12, § 27.

62 Constitution, *supra* note 12, § 27(2).

63 TAC, *supra* note 59; *Soobramoney v. Minister of Health, KwaZulu-Natal*, 1998 (1) SA 765 (CC) (S. Afr.).

64
*Id*. §§ 9–10.

65
*Government of the Republic of South Africa v. Grootboom*, 2001 (1) SA 46 (CC) (S. Afr.).

66 Constitution, *supra* note 12, §§ 27–28(1).

67 Soobramoney, *supra* note 63.

68
*Surrogacy Advisory Group v. Minister of Health*, 2023 (1) SA 241 (GP) (S. Afr.).

69 Constitution, *supra* note 12, § 27.

70 TAC, *supra* note 59.

71 Surrogacy Advisory Group, *supra* note 68.

72 Constitution, *supra* note 12, § 16(1)(d).

73 D. Thaldar & M. Steytler, *Time for Cinderella to Go to the Ball: Reflections on the Right to Freedom of Scientific Research*, 138 S. Afr. Law J. 260 (2021).

74
*South African National Defence Union v. Minister of Defence*, 1999 (4) SA 469 (CC) (S. Afr.).

75
*Islamic Unity Convention v. Independent Broadcasting Authority*, 2002 (4) SA 294 (CC) (S. Afr.).

76 E.S. Lander et al., *Adopt a moratorium on heritable genome editing*, 567 NATURE 165 (2019).

77
*Laugh It Off Promotions CC v. South African Breweries Int’l (Finance) BV t/a Sabmark Int’l*, 2006 (1) SA 144 (CC) (S. Afr.).

78 Thaldar & Steytler, *supra* note 73.

79 Lander et al., *supra* note 76.

80 Constitution, *supra* note 12, § 16(1)(d).

81 Thaldar & Steytler, *supra* note 73.

82 South African National Defence Union, *supra* note 74.

83
*Islamic Unity Convention v. Independent Broadcasting Authority and Others*, *supra* note 75.

84 Constitution, *supra* note 12, § 16(1)(d).

85
*Id*.

86 Lander et al., *supra* note 76.

87 Thaldar & Steytler,  *supra* note 73.

88 Constitution, *supra* note 12, § 12(2).

89
*Id*. § 16(1)(d).

90 Thaldar & Steytler,  *supra* note 73.

91 Constitution, *supra* note 12, § 27.

92
*Id.*

93 TAC, *supra* note 59; Constitution, *supra* note 12, § 27.

94 Surrogacy Advisory Group, *supra* note 68.

95
*Id.*

96 D. Thaldar, B. Shozi & T. Kamwendo, *Culture and context: Why the Global Discourse on Heritable Genome Editing Should Be Broadened From the South African Perspective*, (4) BioLaw J 409 (2021); Thaldar et al., *supra* note 14; Kamwendo, *supra* note 4.

97 Constitution, *supra* note 12, § 27.

98 Thaldar et al., *supra* note 13.

99 J. Abelson, P.G. Forest, J. Eyles, P. Smith, E. Martin & F.-P. Gauvin, *Deliberations About Deliberative Methods: Issues in the Design and Evaluation of Public Participation Processes*, 57 Soc. Sci. & Med. 239 (2003); M.M. Burgess, *From ‘Trust Us’ to Participatory Governance: Deliberative Publics and Science Policy*, 23 Pub. Underst. Sci. 48 (2014).

100
*Id*.

101 Thaldar et al., *supra* note 13.

102 Thaldar et al., *supra* note 96; WHO, *supra* note 42; UNAIDS, *supra* note 43.

103 Thaldar et al., *supra* note 13.

104
*Id.*

105 A. van Mil, H. Hopkins & S. Kinsella, *Potential Uses for Genetic Technologies: Dialogue and Engagement Research Conducted on Behalf of the Royal Society* (Hopkins Van Mil: Creating Connections Ltd (HVM) Dec. 2017).

106 The Associated Press–NORC Center for Public Affairs Research, *Human Genetic Engineering*, https://apnorc.org/projects/human-genetic-engineering/ (accessed Dec. 8, 2021).

107 C. Funk, A. Tyson, B. Kennedy & C. Johnson, *Biotechnology Research Viewed With Caution Globally, but Most Support Gene Editing for Babies to Treat Disease* (Pew Research Center Science & Society, Dec. 10, 2020).

108 NHA, *supra* note 19, §§ 57(1), 57(4).

109 Thaldar et al., *supra* note 96; WHO, *supra* note 42; UNAIDS, *supra* note 43.

110 Thaldar et al., *supra* note 13.

111 Constitution, *supra* note 12, § 16(1)(d).

112 TAC, *supra* note 59.

113 Surrogacy Advisory Group, *supra* note 68.

114 Thaldar et al., *supra* note 13.

115 Van Mil, *supra* note 105; AP-NORC, *supra* note 106; Funk et al., *supra* note 107.

116 Thaldar et al., *supra* note 96; WHO, *supra* note 42; UNAIDS, *supra* note 43.

117 NHREC, *supra* note 9, at s 4.3.2, 74–75.

118 Constitution, *supra* note 12, §§ 16(1)(d), 27.

119 Thaldar et al., *supra* note 13.

120 NHREC, *supra* note 9.

121
*Id*. § 4.3.2(a), at 75.

122
*Id.* § 4.3.2(c), at 75.

123
*Id.* § 4.3.2(b), at 75.

124
*Id.* § 4.3.2(b), at 75.

125
*Id.* § 4.3.2(g), at 75.

126
*Id.* § 4.3.2(g), at 75.

127
*Id*. § 4.3.2(f), at 75.

128
*Id*. § 4.3.2(a), at 75.

129
*Id.*

130 Thaldar et al., *supra* note 13; Thaldar et al., *supra* note 96; WHO, *supra* note 42; UNAIDS, *supra* note 43.

131 Thaldar et al., *supra* note 13.

132 NHREC, *supra* note 9, a§ 4.3.2(b), at 75.

133 Constitution, *supra* note 12, § 10.

134 NHREC, *supra* note 9, 4.3.2(b), at 75.

135
*Id.*

136 NHREC, *supra* note 9, § 4.3.2(c), at 75.

137 Thaldar & Shozi, *supra* note 3.

138 NHREC, *supra* note 9, § 4.3.2(d), at 75.

139 TAC, *supra* note 59.

140 NHREC, *supra* note 9, § 4.3.2(d), at 75.

141 See: Thaldar & Shozi, *supra* note 3.

142 NHREC, *supra* note 9, § 4.3.2(f), at 75.

143 B. Shozi & D. Thaldar, *Promoting Equality in the Governance of Heritable Human Genome Editing through Ubuntu: Reflecting on a South African Public Engagement Study*, 23 Am. J. Bioeth. 43 (2023).

144 Thaldar & Shozi, *supra* note 3.

145 NHREC, *supra* note 9, § 4.3.2(g), at 75; NHA, *supra* note 19, § 57(4).

146 NHA, *supra* note 19, § 57(1).

147 For proposed criteria for embryo research in South Africa, see: D.W. Jordaan, *Criteria for Pre-embryo Research in South Africa: An Analysis Within the Paradigm of Respect for the Pre-embryo*, 27 Med. & L. 417 (2008).

148 NHREC, *supra* note 9, § 4.3.2(a), at 75.

149 NHREC, *supra* note 9, §§ 4.3.2(a), (e), at 75.

150 Constitution, *supra* note 12, § 10.

151
*Id*. § 9.

152
*Id*. § 10.

153
*Id*. § 12.

154
*Id*. § 16(1)(d).

155
*Id*. § 27.

156 NHREC, *supra* note 9, § 4.3.2(e), at 75.

157
*Id.* § 4.3.2(b), at 75.

158
*Id.* § 4.3.2(g), at 75.

159 Constitution, *supra* note 12, § 27.

160 Baylis & Hasson, *supra* note 11; De Vries, *supra* note 11; Ramsay et al., *supra* note 11.

161
*Id.*

162 Baylis & Hasson, *supra* note 11.

163 World Health Org., *WHO Expert Advisory Committee on Developing Global Standards for Governance and Oversight of Human Genome Editing: Report of the Third Meeting, Cape Town, South Africa, 25–26 February 2020* (World Health Org. 2021), https://iris.who.int/bitstream/handle/10665/345252/9789240028494-eng.pdf (accessed Nov. 3, 2025).

164 For a critique on the WHO guidelines from a South African perspective, see: D. Thaldar, & B. Shozi, *An Imbalanced Approach to Governance? An Analysis of the WHO’s Position on Human Genome Editing*, 37 J. Bioeth. 656 (2023).

165 Center for Genetics and Society, *Katie Hasson*, https://www.geneticsandsociety.org/contributor/katie-hasson (accessed Jul. 28, 2025).

166 Baylis & Hasson, *supra* note 11.

167 Baylis & Hasson, *supra* note 11; See, NHA, *supra* note 19, § 57(1).

168 Constitution, *supra* note 12, §§ 10, 16(1)(d), 27.

169 NHREC, *supra* note 9, § 4.3.2(a), at 75.

170 NHREC, *supra* note 9, § 4.3.2(g), at 75.

171 NHREC, *supra* note 9, § 4.3.2(g), at 75.

172 NHREC, *supra* note 9, § 4.3.2(c), at 75.

173 NHREC, *supra* note 9, § 4.3.2(b), at 75.

174 NHREC, *supra* note 9, § 4.3.2(f), at 75.

175 F. Baylis, M. Darnovsky, K. Hasson & T.M. Krahn, *Human Germline and Heritable Genome Editing: The Global Policy Landscape*, 3 Crispr J. 365 (2020).

176 NHA, *supra* note 19.

177 NHA, *supra* note 19, § 57(1).

178 Thaldar et al., *supra* note 13.

179
*Id.*

180 Constitution, *supra* note 12, §§ 16(1)d, 27.

181 Thaldar et al., *supra* note 96; Thaldar et al., *supra* note 13; Kamwendo, *supra* note 4.

182 Baylis et al., *supra* note 175.

183 WHO, *supra* note 163.

184 Thaldar et al., *supra* note 2.

185 De Vries, *supra* note 11.

186 See: NHA, *supra* note 19, § 57(1).

187 Baylis & Hasson, supra note 11.

188 Thaldar et al., *supra* note 13.

189 Constitution, *supra* note 12, §§ 10, 16, 27.

190 NHA, *supra* note 19.

191 Thaldar et al., *supra* note 13.

192 Ramsay et al., *supra* note 11.

193 See: NHA, *supra* note 19, § 57(1).

194 National Science and Technology Forum (NSTF), *Prof Michèle Ramsay*, https://nstf.org.za/2020/09/02/prof-michele-ramsay/ (accessed Aug. 28, 2025).

195 WHO, *supra* note 163.

196 Ramsay et al., *supra* note 11.

197 NHA, *supra* note 19, § 57(1).

198
*Id.*; *Regulations Relating to Artificial Fertilisation of Persons*, GN R.175, GG 35099 (Mar. 2, 2012) (S. Afr.).

199 Ramsay et al., *supra* note 11.

200 D. Thaldar, S. Soni, L. Prinsen, N. Mnyandu, M. Botes & J. Kinderlerer, *In Defence of South Africa’s National Health Research Ethics Council Guidelines on Heritable Human Genome Editing*, 115 S. Afr. Me. J. e2942 (2025).

201 M. Ramsay, M. Pepper, J. de Vries, S. Mahomed & E. Flack-Davison, *Right of Reply – Response to: In Defence of South Africa’s National Health Research Ethics Council Guidelines on Heritable Human Genome Editing*, 115 S. Afr. Med. J. e3083 (2025).

202
*Id.* at 8.

203
*Id.*

204 P.D. Hopkins, *Bad copies. How Popular Media Represent Cloning as An Ethical Problem*, 28 Hastings Cent. Rep. 6 (1998).

205 NHA, *supra* note 19, § 57(1).

206 Ramsay et al., *supra* note 201.

207
*Id.*

208 Thaldar et al., *supra* note 13.

209 Ramsay et al., *supra* note 11.

210 Baylis & Hasson, *supra* note 11.

211 Constitution, *supra* note 12, §§ 16(1)(d), 27.

212 Ramsay et al., *supra* note 11; Ramsay et al., *supra* note 201.

213 See: NHA, *supra* note 19, §§ 57(1).

214 Ramsay et al., *supra* note 11.

215 Baylis & Hasson, *supra* note 11.

216 De Vries, *supra* note 11.

217 Ramsay et al., *supra* note 11.

218 NHREC, supra note 9, § 4.3.2, at 74–75.

219 NHA, *supra* note 19, § 57(1).

220 See: NHREC, *supra* note 9, at ii-iii.

221 NHREC, *supra* note 9, at 74.

222 P. Engel-Hills, *Response by the Chairperson of the National Health Research Ethics Council on the Conversation Piece Titled ‘South Africa Amended Its Research Guidelines to Allow for Heritable Human Genome Editing’* (Oct. 25, 2024) (unpublished communiqué). This document is not accessible online. As such, it is provided as Supplementary Document 2.

223 See: NHA, *supra* note 19, § 57(1).

224 Baylis & Hasson, *supra* note 11.

225 Ramsay et al., *supra* note 201.

226 Southern African Society for Human Genetics (SASHG), *SASHG Committee Consensus Statement on Heritable Human Genome Editing* (Dec. 13, 2024), https://sashg.org/wp-content/uploads/2025/01/SASHG-Committee-Consensus-statement-on-HGGE-Indaba-20241213.pdf (accessed Aug. 28, 2025).

227 Nat’l Health Research Ethics Council, *South African Ethics in Health Research Guidelines – Principles, Processes and Structures* (3d ed., version 3.1, Nat’l Dep’t of Health 2024) at 74.

228 NHREC, *supra* note 9, § 4.3.2(a), 75.

229
*Id.* § 4.3.2(c).

230
*Id.* § 4.3.2(f).

231 S. Mahomed, M. Ramsay, M.S. Pepper, J. de Vries & E. Flack-Davison, *Section on Heritable Human Genome Editing Withdrawn From the National Health Research Ethics Council Guidelines*, 115 S. Afr. Med. J. e3967 (2025).

232 Baylis & Hasson, *supra* note 11.

233 De Vries, *supra* note 11.

234 Ramsay et al., *supra* note 11.

235 Mahomed et al., *supra* note 231.

236
*Id.*

237 NHREC, *supra* note 9, § 4.3.2(g), at 75.

238
*Id.* § 4.3.2(a), at 75.

239
*Id.* §4.3.2(b), at 75.

240
*Id.* § 4.3.2(f), at 75.

241 NHA, *supra* note 19, § 57(4).

242 Mahomed et al., *supra* note 231.

243 NHA, *supra* note 19, § 57(1).

244 See: Baylis & Hasson, *supra* note 11; De Vries, *supra* note 11; Ramsay et al., *supra* note 11.

245 Thaldar et al., *supra* note 13.

246 Mahomed et al., *supra* note 231.

247 NHREC, *supra* note 9.

248 Baylis & Hasson, *supra* note 11; De Vries, *supra* note 11; Ramsay et al., *supra* note 11.

249 Di Caelers, *South Africa Withdraws Genome Editing Guideline Over Fears About ‘Designer Babies’*, Nature Africa, https://www.nature.com/articles/d44148-025-00254-7 (accessed Aug. 28, 2025).

250
*AB v. Minister of Social Development*, 2017 (3) SA 570 (CC) (S. Afr.).

251 Thaldar, *supra* note 5.

