# Supplementary material for: Heritable human genome editing and the politics of law: the South African case study
Source: J Law Biosci. 2025 Dec 23;12(2):lsaf029. doi: 10.1093/jlb/lsaf029 (PMC12722161; doi:10.1093/jlb/lsaf029)
Supplement: lsaf029_Statement_NHREC_heritable_human_genome_editing_November_2024 [file lsaf029_statement_nhrec_heritable_human_genome_editing_november_2024.pdf]

RESPONSE BY THE CHAIRPERSON OF THE NATIONAL HEALTH RESEARCH ETHICS COUNCIL ON THE *CONVERSATION* PIECE (25 OCTOBER 2024) TITLED, "SOUTH AFRICA AMENDED ITS RESEARCH GUIDELINES TO ALLOW FOR HERITABLE HUMAN GENOME EDITING"

The article titled, "South Africa amended its research guidelines to allow for heritable human genome editing", published in *The Conversation* (25 October 2024) has reference.

This article alleges that the National Guidelines on Ethics in Health Research, published earlier this year, appear to permit the use of genome editing in South Africa to create genetically modified children. Heritable human genome editing (HHGE), as rightly stated in the article, raises significant ethical questions, including concerns about eugenics, safety and long-term effects, among others. The article correctly acknowledges that the National Health Act 61 of 2003 prohibits HHGE, yet suggests that the new research ethics guidelines are not aligned with this position.

Firstly, the National Health Research Ethics Council of South Africa (NHREC) wishes to clarify that neither the National Health Act, nor the 2024 National Guidelines on Ethics in Health Research, legalise HHGE for use in therapy in any way. The prohibition in the National Health Act makes this position very clear. The "reproductive cloning of a human being" is prohibited in section 57(1) of the National Health Act, punishable as a criminal office by a fine and up to 5 years' imprisonment. Furthermore, the same section states that the Minister may permit research on stem cells and human fertilised eggs that are less than 14 days old. We believe that "reading in" a permissive approach concerning HHGE to the guidelines is unconvincing but, nevertheless, we will address this potential confusion by clarifying the wording in the 2024 ethical guidelines.

The NHREC concedes that the wording in the guidelines may have caused some confusion and unnecessary alarm. However, it is important to note that the 2024 research ethics guidelines are grounded in a set of key principles that ensure the integrity and ethical conduct of research involving human participants. As a principle-based document, the context of the entire set of guidelines is relevant and we caution against any interpretation that fails to consider the key principles expressed in the guidelines. These ethical guidelines provide a framework for ethical reasoning and decision-making rather than a set of legal rules. As the prohibition of HHGE is stated in the National Health Act, the 2024 research ethics guidelines stipulate with regard to HHGE that South African researchers "must adhere to all relevant laws governing HHGE research" (par 4.3.2(g)). Section 73 of the National Health Act requires every organisation that conducts "health research" to have a health research ethics committee (registered with the NHREC) or have access to a registered health research ethics committee that must review research proposals and protocols, and grant approval where research proposals and protocols meet the prescribed ethical standards; and have oversight on the approved study. The definition of "health research" is broad, meaning that HHGE research would fall within the scope of health research, making ethics approval a legal requirement.

Moreover, two sets of different regulations promulgated in accordance with the Chapters 8 and 9 of the National Health Act have relevance. Since HHGE research would require the donation of germline cells, the 2014 Regulations Relating to Research with Human Participants, listing that further requirements, apply. HHGE research would, in addition need to comply with the 2012 Regulations Relating to the Use of Human Biological Material, which contain further legal requirements for "genetic health research". The nature of HHGE research is innately genetic, making HHGE a form of "genetic health research".

In the final instance, with the Constitution of the Republic of South Africa (1996) as the supreme law in South Africa, the ethico-legal position regarding HHGE should first and foremost be informed by the values of the Constitution, most prominently dignity, equality and freedom. It is therefore unfortunate that members of the research ethics community, including internationally, have chosen to sensationalise a topic that is poorly understood by many.

8 November 2024

PROF PENELOPE ENGEL-HILLS
